# Nurses’ Knowledge and Skills After Use of an Augmented Reality App for Advanced Cardiac Life Support Training: Randomized Controlled Trial

**DOI:** 10.2196/57327

**Published:** 2024-12-05

**Authors:** Wan-Na Sun, Min-Chai Hsieh, Wei-Fang Wang

**Affiliations:** 1 Department of Nursing National Cheng Kung University Hospital College of Medicine, National Cheng Kung University Tainan Taiwan; 2 Department of Nursing College of Medicine National Cheng Kung University Tainan Taiwan; 3 College of Nursing Chung Hwa University of Medical Technology Tainan Taiwan; 4 Tainan University of Technology Tainan Taiwan

**Keywords:** augmented reality, technology intervention, randomized controlled trial, advanced cardiac life support, nursing education

## Abstract

**Background:**

Advanced cardiac life support (ACLS) skills are essential for nurses. During the COVID-19 pandemic, augmented reality (AR) technologies were incorporated into medical education to increase learning motivation and accessibility.

**Objective:**

This study aims to determine whether AR for educational applications can significantly improve crash cart learning, learning motivation, cognitive load, and system usability. It focused on a subgroup of nurses with less than 2 years of experience.

**Methods:**

This randomized controlled trial study was conducted in a medical center in southern Taiwan. An ACLS cart training course was developed using AR technologies in the first stage. Additionally, the efficacy of the developed ACLS training course was evaluated. The AR group used a crash cart learning system developed with AR technology, while the control group received traditional lecture-based instruction. Both groups were evaluated immediately after the course. Performance was assessed through learning outcomes related to overall ACLS and crash cart use. The Instructional Materials Motivation Survey, System Usability Scale, and Cognitive Load Theory Questionnaire were also used to assess secondary outcomes in the AR group. Subgroup analyses were performed for nurses with less than 2 years of experience.

**Results:**

All 102 nurses completed the course, with 43 nurses in the AR group and 59 nurses in the control group. The AR group outperformed the control group regarding overall ACLS outcomes and crash cart learning outcomes (*P*=.002; *P*=.01). The improvement rate was the largest for new staff regardless of the overall learning effect and the crash cart effect. Subgroup analysis revealed that nurses with less than 2 years of experience in the AR group showed more significant improvements in both overall learning (*P*<.001) and crash cart outcomes (*P*<.001) compared to their counterparts in the control group. For nurses with more than 2 years of experience, no significant differences were found between the AR and control groups in posttraining learning outcomes for the crash cart (*P*=.32). The AR group demonstrated high scores for motivation (Instructional Materials Motivation Survey mean score 141.65, SD 19.25) and system usability (System Usability Scale mean score 90.47, SD 11.91), as well as a low score for cognitive load (Cognitive Load Theory Questionnaire mean score 15.42, SD 5.76).

**Conclusions:**

AR-based learning significantly improves ACLS knowledge and skills, especially for nurses with less experience, compared to traditional methods. The high usability and motivational benefits of AR suggest its potential for broader applications in nursing education.

**Trial Registration:**

ClinicalTrials.gov NCT06057285; https://clinicaltrials.gov/ct2/show/NCT06057285

## Introduction

### Challenges in Advanced Cardiac Life Support Training and Nurse Preparedness

Advanced cardiac life support (ACLS) is an essential and indispensable nursing skill. A nurse with adequate ACLS skills can provide a patient with comprehensive first aid treatment in an emergency, avoiding missing the “golden hour” of treatment [1]. In-hospital cardiac arrest (IHCA) affected 292,000 patients in the United States between 2008 and 2017 each year, equivalent to IHCA occurring in nearly 10 out of every 1000 inpatients [2]. The IHCA incidence rate increased sharply during the COVID-19 pandemic, with a 30-day survival rate after first aid of merely 25% [2,3]. In Taiwan, 957 incidents of unexpected IHCA occurred in 2020, accounting for 1.2% of all patient safety incidents that year and ranking first among acute life-threatening events for patients (51.1%) [4]. During an IHCA, nurses are generally responsible for determining and providing appropriate first aid measures. Therefore, training and enhancing ACLS skills among nurses is crucial. A lack of experience, knowledge, and familiarity with ACLS cart–related skills may have contributed to the underperformance of nurses delivering first aid [5,6]. In first aid scenarios, nurses who lack clinical experience generally experience immense stress due to the immediacy and imminence of patient rescue. Chen and Tzeng [7] surveyed 164 nurses with less than 2 years of experience in terms of their knowledge of ACLS medications and perceived stress during the delivery of ACLS and found that 94% of the nurses experienced immense stress delivering first aid. The stress was more significant for those with less knowledge of ACLS medications (*P*=.002). Moreover, Chen and Tzeng [7] reported that only 6% of the nurses were adequately trained through ACLS training programs, and the best time to implement relevant training programs was deemed by 41.8% of nurses between 3 and 6 months after starting work.

### Challenges in Traditional ACLS Training

Current ACLS training programs in Taiwan are generally conducted through experience-sharing, case discussions, or bedside teaching, which is challenging for nurses with insufficient clinical experience and does not give every nurse a chance to practice [1]. Furthermore, traditional teaching methods were suspended or hindered in 2019 due to the COVID-19 pandemic. Clinical ACLS education should focus on practical learning and teaching material accessibility. The scientific statement titled “Resuscitation Education Science: Educational Strategies to Improve Outcomes from Cardiac Arrest,” published by the American Heart Association, mentions that immersive technologies and gamified learning effectively enhance learning outcomes [8]. The attention, relevance, confidence, and satisfaction model of learning motivation is a teaching strategy model proposed by Keller [9,10] in 1987. An instructional process based on this model first draws the learner’s attention, demonstrates relevance with them through personal or relevant experience, and elevates his or her confidence during learning, thereby achieving satisfaction. For Keller [9,10], maintaining attention is crucial. The development of medical technology has trended toward integrating artificial intelligence into medical systems. The COVID-19 pandemic further acted as a catalyst for the incorporation of augmented reality (AR), virtual reality (VR), and even mixed reality into medical education, in which relevance is demonstrated to the learner through actual and virtual reality simulated scenarios, enabling them to learn independently without the constraints of location, time, and other factors, thereby enhancing their confidence. Learner satisfaction is increased through repeatable practice beyond time and location constraints [11].

### Trends in the Adoption of AR in Health Care

The AR industries are not industries of emerging technologies, and their flourishing development in recent years into various domains has been catalyzed through their progressive technological maturation and the COVID-19 pandemic. Application of AR technologies in health care has increased by 25% over the past 3 years, and in 2028, the annual economic output value was estimated to exceed US $9.5 billion [12]. AR involves superimposing virtual information onto an actual environment through a mobile device, with the user viewing the virtual image and data in real-life scenarios shown on the mobile device’s screen; AR is characterized by a combination of interaction between the real world and virtual information [13,14]. AR is more valuable than VR for health care education. VR involves computer-based simulation of real-life scenarios in which the user interacts with and views the simulation through a device. Therefore, VR requires a headset and another person’s assistance while immersed in a virtual environment. Furthermore, VR has been associated with physical discomforts, such as dizziness, nausea, vomiting, and sore eyes, that can affect or interrupt the learning process and decrease learning motivation [14]. AR systems are more portable than VR systems. AR can be operated using a simple mobile device, such as a cellphone or a tablet, increasing the accessibility of teaching materials and equipment and allowing drills to be conducted through real-life scenarios and images. While mobile-based AR offers the convenience of accessibility through smartphones or tablets, it is essential to note that AR technology is not limited to mobile devices. AR can also be performed using head-mounted displays (HMDs), which provide immersive experiences but are typically not considered “mobile” in the same sense as smartphones or AR glasses. AR systems can be delivered through various devices, including mobile-based platforms such as smartphones or AR glasses, which are generally more portable than VR systems. However, AR can also be performed using HMDs, which offer a more immersive experience but are typically less portable than mobile devices. Mobile-based AR and HMDs offer unique advantages depending on the training context. Mobile-based AR and HMD-based AR offer unique advantages depending on the context of their use, with mobile-based AR being more accessible for everyday learning environments and HMDs offering enhanced immersion for more specialized training [15]. In health care education, drills tend to be conducted based on the operation of related technologies. Many studies have indicated that learning materials developed using AR technology can effectively enhance participants’ performance and improve focus. This is especially important in clinical work settings, where simulations are required. AR technology allows learners to study independently, significantly improving learning outcomes [11].

The studies have demonstrated that education on ACLS nursing skills is an important issue, and an appropriate model of learning motivation should first trigger the learner’s motivation and enable them to identify the relevance of the content learned. This study used AR technologies to develop a first-aid cart learning system. The accessibility of mobile devices enables learners to surpass location and time constraints and learn by relating to crash cart–related actual clinical images and sites, thereby enhancing their confidence and increasing their satisfaction with acquiring ACLS knowledge and skills.

## Methods

### Study Design, Participants, and Setting

**Figure 1:**
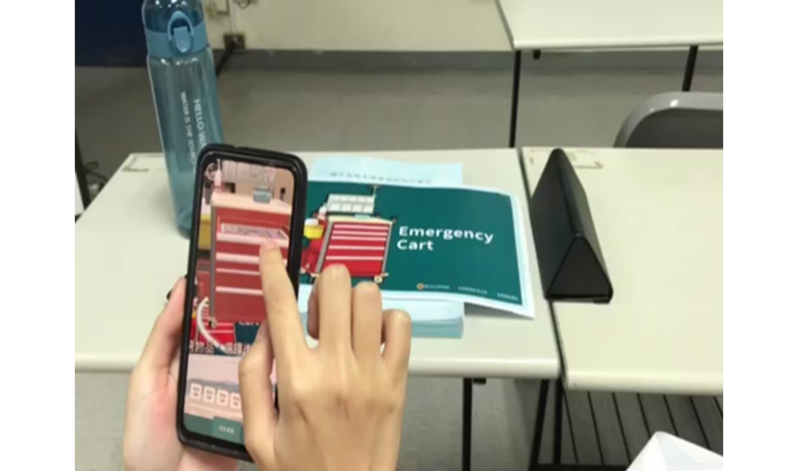
Actual operation process.

This interventional and experimental study was registered on ClinicalTrials.gov (NCT06057285). A single-blinded research method was adopted, and participants were recruited through convenience sampling. The research setting was a medical center in southern Taiwan. All nurses were required to have a university degree as part of their work at the institution. This study was divided into 2 stages. In the first stage, a crash cart learning system was developed using AR technologies, and to enhance learning accessibility, necessary teaching materials for ACLS drills (ie, the structure and content of a crash cart) were digitized to enable the operation of this system on mobile devices (Figure 1).

The crash cart learning system uses AR technology to transform a physical crash cart into a virtual teaching tool comprising five parts. The first part includes 13 emergency medications, detailing their effects, side effects, and dosages. The second part covers infusions, such as normal saline, Dextrose 5%, lactated Ringer’s solution, and blood draw supplies. The third part features intubation equipment, including various sizes of endotracheal tubes. The fourth part covers tracheostomy tubes, laryngeal mask airways, and central venous catheters. The fifth part covers Ambu bags and suction devices. In addition to simulating a physical crash cart, the system provides instructions for each item.

**Figure 2:**
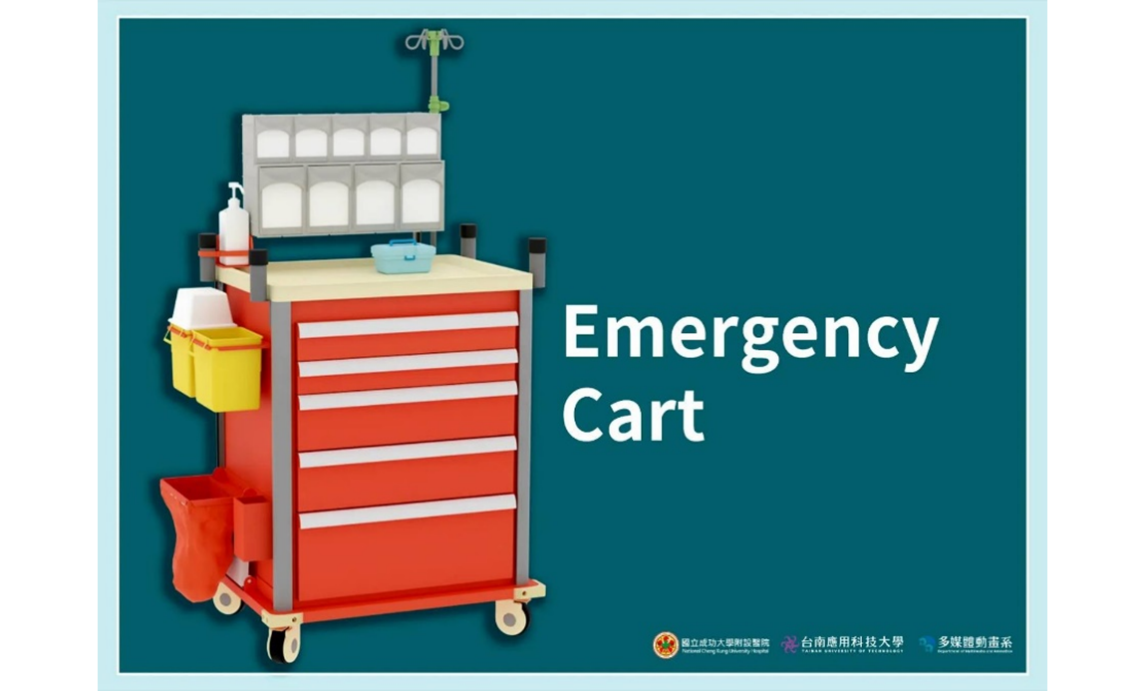
AR marker of the crash cart. The AR marker image opens the crash cart learning system when scanned with a mobile device.

In the second stage, the developed crash cart learning system was applied to ACLS drills in a regular ACLS training program, and an AR group used this learning system to assist in their learning. The AR group scanned the dedicated AR marker using a mobile device (Figure 2) to learn about emergency medications, intubation equipment, and other tools. In contrast, the control group received instruction through lectures. Regardless of the group, the learning content included using emergency drugs, side effects and dosage, intubation, infusion, and ACLS procedures. The same lecturer and teaching assistant conducted it, and each class lasted about one hour. After the class, the learning effectiveness was evaluated immediately.

### Participants

Nurses aged 20 years or older, who could speak and read Mandarin; worked in intensive care units, emergency rooms, or general wards; and participated in resuscitation processes were recruited. All participants completed the questionnaire after the research objective was explained. Nurses working in pediatric departments, as well as nursing supervisors or part-time nurses, were excluded.

A priori power analysis was conducted using G*Power (Heinrich-Heine-Universität Düsseldorf). The sample size (n=82) was obtained using a power level of 0.8, an α level set at .05, and a small to medium effect size of 0.3 [16,17]. The estimated dropout rate was 20%, and the estimated number of persons to be recruited was 99.

### Instrumentation

The questionnaire used in this study was rated by 3 experts (Bo-Shen Chen, Jhen-Yu Ji, and Shu-Jhen Su), all of whom have at least 10 years of experience in their professional fields. The experts were a clinical teacher, a departmental teaching nurse, and a senior cardiopulmonary resuscitation (CPR) instructor rated as excellent by the education center for this study in 2019. The experts assessed the appropriateness, relevancy, and clarity of the questionnaire content, giving scores of 1 (inappropriate and to be deleted), 2 (appropriate but requiring vast amendment), 3 (appropriate but requiring slight adjustment), and 4 (extremely appropriate). After the assessment, items with a score of ≥3 were included in the calculation of the content validity index (CVI), and items with a score of <3 were amended or deleted.

### Learning Outcomes Questionnaire

To measure the outcomes of the ACLS drills using AR-based teaching aids, this study developed a learning outcome questionnaire. The questionnaire was formulated according to the latest version of the *Advanced Cardiovascular Life Support Provider Manual*. To increase its reliability and validity, an open call was conducted to choose 3 experienced CPR instructors with a minimum of 5 years of experience. The items were classified as easy (20%), intermediate (50%), and difficult (30%). The CPR instructors created 25 questions, including 10 items that were based on the content of the crash cart learning system. The total score ranged from 0 to 100, with a higher score indicating better learning effectiveness. The appropriateness of the finalized learning outcome test was evaluated by 2 experienced CPR instructors other than those who created the items. This study demonstrated content validity with an item-CVI ranging from 0.88 to 0.95.

### Instructional Materials Motivation Survey

The Instructional Materials Motivation Survey (IMMS) was developed, derived from the attention, relevance, confidence, and satisfaction model proposed by Keller [9,10]. It comprises 36 items, including 26 positively and 10 negatively worded items. It consists of 4 subscales, namely the attention subscale (12 items), relevance subscale (9 items), confidence subscale (9 items), and satisfaction subscale (6 items). Items are rated on a 5-point Likert scale with end points ranging from 1 (strongly disagree) to 5 (strongly agree), and the total score ranges between 36 and 180. Higher scores indicate stronger learning motivation. The scale exhibited good internal reliability, as evidenced by Cronbach α coefficients ranging from 0.81 to 0.96, and it also demonstrated content validity with a CVI of 0.85 [9].

### System Usability Scale

Brooke used the System Usability Scale (SUS) in 1986 to test the overall usability of a product. The SUS measures a user’s perception of use and comprises ten items, including five positively worded items and five negatively worded items, which are rated on a 5-point Likert scale with endpoints ranging from 1 (strongly disagree) to 5 (strongly agree). The total score was multiplied by 2.5 to obtain a final score, which ranged between 25 and 100, with higher total scores indicating higher satisfaction with the system and total scores <68 indicating inferior system usability [18]. The scale has exhibited good internal reliability, as evidenced by Cronbach α coefficient of 0.84, and it has also demonstrated content validity with a CVI of 0.97 [19,20].

### Cognitive Load Theory Questionnaire

The cognitive load theory (CLT) proposed by Sweller et al [21] comprises endogenous and exogenous factors. Endogenous factors refer to the learner per se and are associated with their effort and mental ability. In contrast, exogenous factors refer to the external environment, such as teaching materials and environments. The Cognitive Load Theory Questionnaire contains 8 items rated on a 5-point Likert scale with endpoints ranging from 1 (strongly disagree) to 5 (strongly agree) and total scores ranging between 8 and 40. Higher scores indicate a more significant cognitive load perceived by the learner for the item. The scale has exhibited good internal reliability, as evidenced by Cronbach α coefficients ranging from 0.88 to 0.93, and it has also demonstrated content validity with a CVI of 0.96 [21,22].

### Ethical Considerations

Before the second stage, this study was approved by the Institutional Review Board of the National Cheng Kung University Hospital (approval A-ER-109-383). Before the study commenced, the research plan was presented to the unit supervisors, and the study procedures were clearly explained to all participants. Participation in the study was entirely voluntary, and nonparticipation did not affect the participants’ job duties or educational opportunities. Informed consent was obtained from all participants before enrolling in the study, and each participant signed a consent form. To ensure the privacy and confidentiality of the participants, all collected data were deidentified using numerical codes, and no personally identifiable information was linked to the data. The deidentified data were securely stored in a locked drawer accessible only to the research team. No monetary or other forms of compensation were provided to the participants for their involvement in the study.

### Data Collection

Participants were recruited from 4 units with similar numbers of CPR deliveries, selected through the hospital’s provided database and assigned using convenience sampling. Additionally, participants were grouped using convenience sampling in 2 consecutive ACLS training courses organized by the hospital for new nurses. This is different from the general ACLS course. It is a training course for all new nurses in the hospital. The drugs and tools used are all from the hospital, which increases the new nurses’ familiarity with the hospital. The ACLS course was conducted after the research objective and course outline were presented to related executives and the participants and after the participants provided written consent.

The participants of both groups completed a learning outcome test before and after learning immediately by trained research assistants who were not involved in the training sessions. The participants of the AR group completed the IMMS, SUS, and Cognitive Load Theory Questionnaire the day after the course, which took approximately 10 minutes to complete.

### Data Analysis

Database construction and statistical analysis were performed in SPSS (version 20.0; IBM Corp). A *P* value of <.05 was considered statistically significant. Descriptive statistics (numbers, percentages, means, and SD) were used to summarize the participants’ demographic characteristics and item scores. The participants’ essential attributes were tested for homogeneity using the independent 2-tailed *t* test and chi-square test. Moreover, descriptive statistics and 2-tailed *t* tests were conducted to investigate the effects of the crash cart learning system for ACLS training on nurses’ performance in terms of learning effectiveness, motivation, system usability, and cognitive load. The government has implemented the nurse postgraduate year program in Taiwan to help nursing students transition after graduation. This program focuses on intensive training during the first 2 years of postgraduate study [23], aiding their adaptation to clinical work. Therefore, this study includes a specific subgroup analysis of nurses with less than 2 years of experience.

## Results

### Demographic Characteristics

**Figure 3:**
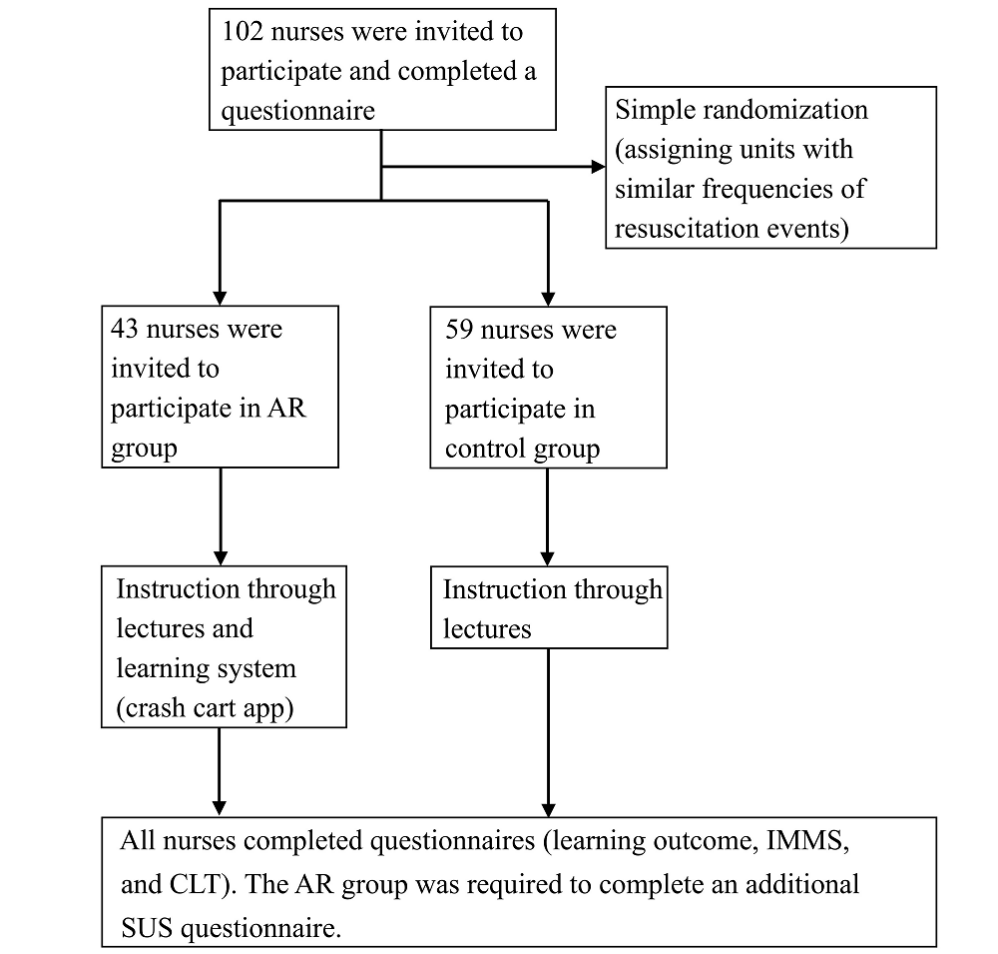
Study enrollment flowchart. AR: augmented reality; CLT: Cognitive Load Theory; IMMS: Instructional Materials Motivation Survey; SUS: System Usability Scale.

Data were collected between August and December 2021. No participants dropped out. In total, 102 nurses completed the course, with 43 nurses in the AR group and 59 nurses in the control group. For an overview of participant flow, see the CONSORT (Consolidated Standards of Reporting Trials) diagram (Figure 3). The average age of the nurses was 26.18 (SD 5.88) years. Women formed the majority (n=95, 93.1%). Among the departments nurses worked in, nurses working in internal medical wards comprised the majority (n=59, 57.8%). A total of 63.7% (n=65) of the nurses had less than 2 years of work experience. Most nurses were ranked N-level (n=66, 64.7%; the nursing ladder in Taiwan is divided into 5 levels: N, N1, N2, N3, and N4, with higher levels indicating greater proficiency) and had an ACLS certificate (n=71, 69.6%). No significant differences in demographic characteristics were observed between the 2 groups (Table 1).

**Table 1:** Demographic data of the sample.

			All (n=102)		AR^a^ group (n=43)		Control group (n=59)		*P* value	
Age (years), mean (SD)			26.18 (5.88)		27.12 (6.63)		25.49 (5.21)			
**Gender, n (%)**										.45
	Men	7 (6.9)		4 (9)		3 (5)				
	Women	95 (93.1)		39 (91)		56 (95)				
**Department, n (%)**										.40
	Internal medical	59 (57.8)		19 (44)		40 (68)				
	Surgery	13 (12.7)		5 (12)		8 (14)				
	Integrated branch	15 (14.7)		11 (26)		4 (7)				
	Emergency	15 (14.7)		8 (19)		7 (12)				
**Work experience, n (%)**										.68
	< 2 years	65 (63.7)		26 (61)		39 (66)				
	2-6 years	24 (23.6)		11 (26)		13 (22)				
	>6-8 years	13 (12.7)		6 (14)		7 (12)				
**Nursing ladder^b^, n (%)**										.51
	N	66 (64.6)		24 (56)		42 (71)				
	N1-N2	27 (26.5)		13 (30)		14 (24)				
	>N3	9 (8.9)		6 (14)		3 (5)				
**ACLS^c^ licenses, n (%)**										.67
	Yes	71 (69.6)		31 (72)		40 (38)				
	No	31 (30.4)		12 (28)		19 (32)				

^a^AR: augmented reality.

^b^The nursing ladder in Taiwan is divided into 5 levels: N, N1, N2, N3, and N4. Nurses are promoted based on their caregiving, teaching, research, and administrative abilities, with higher levels indicating greater proficiency.

^c^ACLS: advanced cardiac life support.

### Learning Effectiveness

For the overall learning outcomes in the AR group, the mean score was 61.12 (SD 14.72) before learning and 74.23 (SD 13.22) after learning, and the difference between overall outcomes before and after learning was significant (*P*<.001). For the control group, the mean score was 58.24 (SD 14.31) before learning, and 64.54 (SD 16.21) after learning, and the difference in the overall learning outcomes before and after learning was also significant (*P*<.001). The difference in learning outcomes between the 2 groups after learning was compared. The AR group outperformed the control group, and the difference was significant (*P*=.002; Table 2).

In Taiwan, new nurses undergo 2 years of training. Therefore, a subgroup analysis was conducted in which nurses were divided according to whether they had 2 years of experience. The mean score of the overall learning outcomes of the nurses of the AR group who had less than 2 years of experience was 52.77 (SD 10.25) before learning, and 68.31 (SD 12.41) after learning, and the difference before and after learning was significant (*P*<.001). For the overall learning outcomes of the nurses of the control group who had less than 2 years of experience, the mean score was 52.56 (SD 11.89) before learning, and 56.92 (SD 11.73) after learning, and the difference between learning outcomes before and after learning was significant (*P*=.01). The difference in learning outcomes after learning between the 2 groups of nurses with less than 2 years of experience was evaluated. The AR group outperformed the control group, and the difference was significant (*P*<.001).

**Table 2:** Learning effectiveness of the 2 groups.

			AR^a^ group							Control group									*P* value^b^
			Pretest, mean (SD)		After test, mean (SD)		*P* value^c^		Pretest, mean (SD)				After test, mean (SD)	*P* value^c^					
**All nurses^d^**																			
	Learning outcome	61.12 (14.72)		74.23 (13.22)		<.001		58.24 (14.31)				64.54 (16.21)			<.001		.002		
	Learning outcome of crash cart	60.00 (22.99)		75.12 (20.75)		<.001		54.75 (23.88)				59.49 (27.32)			.02		.01		
**New nurses^e,f^**																			
	Learning outcome	52.77 (10.25)		68.31 (12.41)		<.001					52.56 (11.89)	56.92 (11.73)				.01	<.001		
	Learning outcome of crash cart	46.54 (16.48)		68.46 (21.11)		<.001					43.59 (18.57)	46.15 (21.96)				.34	<.001		
**Experienced nurses^g,h^**																			
	Learning outcome	73.88 (10.87)		83.29 (8.51)		.001		69.30 (12.14)				79.40 (13.25)			<.001		.80		
	Learning outcome of crash cart	80.59 (14.78)		85.29 (15.86)		.20		76.50 (17.25)				85.50 (15.38)			.001		.32		

^a^AR: augmented reality.

^b^Between group *P* value.

^c^Within-group *P* value.

^d^For all nurses, n=43 for the AR group and n=59 for the control group.

^e^For new nurses, n=26 for the AR group and n=39 for the control group.

^f^Work experience of fewer than 2 years.

^g^For nurses, n=17 for the AR group and n=20 for the control group.

^h^Work experience of more than 2 years.

Among nurses with less than 2 years of experience, in the AR group, the mean score was 52.77 (SD 10.25) before learning and 68.31 (SD 12.41) after learning, and the difference between overall outcomes before and after learning was significant (*P*<.001). For the control group, the mean score was 52.56 (SD 11.89) before learning, and 56.92 (SD 11.73) after learning, and the difference in the overall learning outcomes before and after learning was also significant (*P*=.01). The difference in learning outcomes between the 2 groups after learning was compared. The AR group outperformed the control group, and the difference was significant (*P*<.001; Table 2). Among nurses with more than 2 years of experience, in the AR group, the mean overall learning outcomes score was 73.88 (SD 10.87) before learning and 83.29 (SD 8.51) after learning, and the difference was significant (*P*=.001). In the control group, the mean score was 69.30 (SD 12.14) before learning and 79.40 (SD 13.25) after learning, and the difference was significant (*P*<.001). The difference in learning outcomes after the course between the 2 groups of nurses with more than 2 years of experience was evaluated, and there was no significant difference (*P*=.80; Table 2).

### Learning Effectiveness for the Crash Cart

Of the 25 questions in the learning outcomes test, 10 items addressed the content of a crash cart. For the overall learning outcomes of a crash cart in the AR group, the mean score was 60.00 (SD 22.99) before learning and 75.12 (SD 20.75) after learning, and the difference between learning outcomes for the crash cart before and after learning was significant (*P*<.001). For the control group, the mean score was 54.75 (SD 23.88) before learning and 59.49 (SD 27.32) after learning, and the difference in the overall crash cart learning outcomes before and after learning was also significant (*P*=.02). The difference between the 2 groups in content learning outcome for the crash cart scores after learning was significant (*P=*.01; Table 2).

For nurses with under 2 years of experience, for the AR group, the average crash cart learning outcomes score increased from 46.54 (SD 16.48) before learning to 68.46 (SD 21.11) after learning, showing a significant improvement (*P*<.001). In the control group, the mean score rose from 43.59 (SD 18.57) to 47.15 (SD 21.96) after learning. Still, there was no significant difference (*P*=.34). A comparison of postlearning crash cart outcomes between the 2 groups of nurses with less than 2 years of experience revealed a significant difference (*P*<.001; Table 2). Among nurses who have more than 2 years of experience, In the AR group, the average crash cart learning outcomes score was 80.59 (SD 14.78) before learning and 85.29 (SD 15.86) after learning, with no significant difference (*P*=.20). In contrast, the control group had a mean score of 76.50 (SD 17.25) before learning and 85.50 (SD 15.35) after learning, showing a significant improvement (*P*=.001). There was no significant difference when comparing the post learning crash cart outcomes between the 2 groups of nurses with over 2 years of experience (*P*=.32; Table 2).

### Motivation, Usability, and Cognitive Load Assessment

For the AR group, the mean scores of the IMMS, SUS, and Cognitive Load Theory Questionnaire were 141.65 (SD 19.25), 90.47 (SD 11.91), and 15.42 (SD 5.76), respectively. Specifically, for the IMMS, the mean scores for the attention, relevance, confidence, and satisfaction subscales were 47.84 (SD 6.94), 35.21(SD 5.39), 35.81 (SD 5.09), and 22.79 (SD 3.20), respectively (Table 3).

**Table 3:** The mean score distribution in IMMS^a^, SUS^b^, and CLTQ^c^ in the AR^d^ group.

			Total		Mean (SD)
**IMMS**			36-180		141.65 (19.25)
	Attention	12-60		47.84 (6.94)	
	Relevance	9-45		35.21 (5.39)	
	Confidence	9-45		35.81 (5.09)	
	Satisfaction	6-30		22.79 (3.20)	
SUS			25-100		90.47 (11.91)
CLT			8-40		15.42 (5.76)

^a^IMMS: Instructional Materials Motivation Survey.

^b^SUS: System Usability Scale.

^c^CLTQ: Cognitive Load Theory Questionnaire.

^d^AR: augmented reality.

## Discussion

### Principal Findings

One of the strengths of this study is that all participants were university graduates, and there were no significant demographic differences in work experience or ACLS certification between the control and intervention groups. This homogeneity minimized potential confounding factors, allowing us to attribute the observed performance improvements in the AR group primarily to the intervention rather than differences in participants’ prior knowledge or experience.

### Effectiveness of AR-Based Learning for ACLS Training

This study found that all nurses significantly improved after completing the ACLS training course (*P*=.002). However, the crash cart learning system developed using AR technology demonstrated significantly better learning outcomes than traditional teaching methods. Chen and Liou [24] developed a teaching intervention that involved AR technologies. A total of 95 junior nursing students who were learning how to handle airway obstruction during the delivery of ACLS participated in the study. The results revealed that AR technologies improved learning outcomes, confidence, and satisfaction more than traditional teaching methods [24]. Qualitative interviews revealed an emergency airway obstruction training system using AR technology, and focus group interviews were conducted with 82 nursing students. The results showed that AR technology helped them understand real clinical scenarios better and reduced their learning pressure. Unlike traditional teaching methods, which require following the pace of others, AR allowed them to practice independently and repeatedly after the course, based on their individual needs [1]. However, the crash cart learning system developed using AR technology can enhance participants’ performance. It can be inferred that because the AR system allows for practicing with virtual objects in real-world scenarios, it overcomes the limitations of traditional teaching methods, which lack the ability to simulate emergency clinical situations. By using AR to simulate emergency clinical scenarios, learners experience reduced stress when faced with real-life emergencies [25]. Additionally, this study uses AR marker technology on mobile devices, allowing learners to access the crash cart learning system by simply scanning with their phones. This eliminates the need for specific devices or designated spaces for practice, thereby increasing the accessibility of learning.

### Impact on Nurses With Different Levels of Experience

This study found that nurses with work experience of fewer than 2 years showed significantly improved learning outcomes (*P*<.001) when using AR technology for virtual simulation learning, particularly in crash cart learning. Traditional teaching methods, even using pictures, descriptions, or videos, made it difficult for less experienced nurses to visualize the scenarios. Interestingly, the study also found that for more experienced nurses, there was no significant difference in learning outcomes before and after using the AR-based crash cart learning system (*P*=.20). In contrast, traditional teaching methods yielded significant differences in both ACLS (*P*<.001) and crash cart learning (*P*=.001) for work experience of more than 2 years. Balian et al [8] developed a CPR training system that integrated AR technologies. Their study involved 51 hospital employees. Nurses with an average of 5 years of experience achieved more favorable learning outcomes in complicated and changing ACLS scenarios than participants with less experience. In addition to another study involving 30 cardiopulmonary cerebral resuscitation instructors, it was found that learning through AR techniques was more appealing to inexperienced participants, helping them stay focused during their learning. AR also mitigated the adverse effects of their lack of experience, which often hindered their learning performance, by providing virtual objects overlaid onto real-world environments. This feature reduced inexperienced participants’ challenges, allowing them to grasp concepts better and improve their learning effectiveness [26]. On the other hand, more experienced learners were not as accustomed to using mobile devices for learning. Instead, those with extensive work experience preferred traditional teaching methods, favoring interaction and discussion with instructors to analyze complex clinical scenarios [26]. This prompts us to reflect that learning through AR technology cannot fully address all participants’ needs. In addition to continuously optimizing the variability of simulated scenarios, the learning process should incorporate discussions with instructors to cater to the diverse needs of participants.

### Enhancing Motivation and Reducing Cognitive Load Through AR

The AR technologies in this study did not burden the nurses in terms of system operation and cognitive load. The AR technologies stimulated and maintained learning motivation through mobile devices that were readily accessible to the nurses. A systematic review of students’ attitudes, effectiveness, and motivation in using AR for learning analyzed 28 studies from 2016 to 2023. The results showed that students had more positive learning attitudes when using AR (*P*<.001), and their learning outcomes were better compared to students who did not use AR technology (*P*<.01). However, AR-assisted learning did not improve learning motivation (*P*=.12). The study noted that AR-assisted learning systems are often disrupted by issues such as learning time, internet connectivity, and device problems, which can interrupt the learning process [1].

Regarding system usability, the average score in this study was 90.47 (SD 11.91), indicating that the crash cart learning system is highly usable. According to Choi et al [18], to better quantify the score range, the scale was divided into 5 levels: a score of 90-100 is an A, indicating extremely useful; 80-89 is a B, indicating very useful; 70-79 is a C, indicating good; 60-69 is a D, indicating acceptable; and 0-59 is an F, indicating poor. Based on this study’s score, the system falls into the A level, indicating that participants found the crash cart learning system extremely easy to use.

### Limitation

This study’s limitation is that the teaching course based on the developed crash cart learning system focused on the functions and operation of the crash cart without providing opportunities for nurses to engage in emergency clinical scenarios. This lack of integration with actual ACLS scenarios limits the overall learning experience in first aid training. Future system updates could address this by adding emergency scenarios to enhance the connection between the system and practical emergency response training.

### Conclusions

This study was conducted in a medical center where all nurses must be university graduates and have completed ACLS certifications during their employment. These stringent requirements may have contributed to an artificially higher learning effectiveness, as the participants may have had a more robust foundational knowledge and prior experience. As a result, the findings may not be fully generalizable to settings where nurses have lower requirements.

## Appendix

Multimedia Appendix 1 CONSORT-eHEALTH checklist (V 1.6.2).

## Abbreviations

ACLS: advanced cardiac life support
AR: augmented reality
CLT: cognitive load theory
CPR: cardiopulmonary resuscitation
CVI: content validity index
HMD: head-mounted display
IHCA: in-hospital cardiac arrest
IMMS: Instructional Materials Motivation Survey
SUS: System Usability Scale
VR: virtual reality

## Data Availability

The data sets generated during this study are not publicly available to protect student privacy but are available from the corresponding author upon reasonable request.
