# Supplementary material for: Nurses’ Knowledge and Skills After Use of an Augmented Reality App for Advanced Cardiac Life Support Training: Randomized Controlled Trial
Source: J Med Internet Res. 2024 Dec 5;26:e57327. doi: 10.2196/57327 (PMC11659687; doi:10.2196/57327)
Supplement: Multimedia Appendix 1 [file jmir_v26i1e57327_app1.pdf]

CONSORT-EHEALTH Checklist V1.6.2 Report	Manuscript Number	57327
**(based on CONSORT-EHEALTH V1.6), available at [http://tinyurl.com/consort-ehealth-v1-6].**		
**Date completed**		
11/26/2024 23:41:48		
**by**		
Wan-Na Sun		
Nurses’ Knowledge and Skills After Use of an Augmented Reality App for Advanced Cardiac Life Support Training: Randomized Controlled Trial		
TITLE		
**1a-i) Identify the mode of delivery in the title**		
Nurses’ Knowledge and Skills After Use of an Augmented Reality App for Advanced Cardiac Life Support Training: Randomized Controlled Trial		
**1a-ii) Non-web-based components or important co-interventions in title**		
**1a-iii) Primary condition or target group in the title**		
Nurses’ Knowledge and Skills After Use of an Augmented Reality App for Advanced Cardiac Life Support Training: Randomized Controlled Trial		
ABSTRACT		
**1b-i) Key features/functionalities/components of the intervention and comparator in the METHODS section of the ABSTRACT**		
Nurses’ Knowledge and Skills After Use of an Augmented Reality App for Advanced Cardiac Life Support Training: Randomized Controlled Trial		
**1b-ii) Level of human involvement in the METHODS section of the ABSTRACT**		
**1b-iii) Open vs. closed, web-based (self-assessment) vs. face-to-face assessments in the METHODS section of the ABSTRACT**		
**1b-iv) RESULTS section in abstract must contain use data**		
**1b-v) CONCLUSIONS/DISCUSSION in abstract for negative trials**		
INTRODUCTION		
**2a-i) Problem and the type of system/solution**		
page 3-4 In the second stage, the developed crash cart learning system was applied to ACLS drills in a regular ACLS training program, and an AR group used this learning system to assist in their learning. The AR group scanned the dedicated AR marker using a mobile device (Figure 2) to learn about emergency medications, intubation equipment, and other tools. In contrast, the control group received instruction through lectures. Regardless of the group, the learning content includes using emergency drugs, side effects and dosage, intubation, infusion, and ACLS procedures. The same lecturer and teaching assistant conduct it, and each class lasts about one hour. After the class, the learning effectiveness will be evaluated immediately.		
**2a-ii) Scientific background, rationale: What is known about the (type of) system**		
page 1-2 in manuscripts(introduction section)		
**Does your paper address CONSORT subitem 2b?**		
p1-3 in manuscripts (introduction section)		
METHODS		
**3a) CONSORT: Description of trial design (such as parallel, factorial) including allocation ratio**		
in Methods section (Study Design Participants and Setting) page 3		
**3b) CONSORT: Important changes to methods after trial commencement (such as eligibility criteria), with reasons**		
in methods section(Participants) page 4		
**3b-i) Bug fixes, Downtimes, Content Changes**		
**4a) CONSORT: Eligibility criteria for participants**		
in methods section(Participants) page 3-4		
**4a-i) Computer / Internet literacy**		
**4a-ii) Open vs. closed, web-based vs. face-to-face assessments:**		
page 5 in manuscripts(data collection)		
**4a-iii) Information giving during recruitment**		
page 5 in manuscripts(data collection, and ethical)		
**4b) CONSORT: Settings and locations where the data were collected**		
page 3 in manuscripts(The research setting was a medical center in southern Taiwan. All nurses were required to have a university degree as part of their work at the institution.)		
**4b-i) Report if outcomes were (self-)assessed through online questionnaires**		
page 4-5 in manuscripts(we use paper questionnaire in this study)		
**4b-ii) Report how institutional affiliations are displayed**		
national cheng kung university hospital for data collection		
**5) CONSORT: Describe the interventions for each group with sufficient details to allow replication, including how and when they were actually administered**		
**5-i) Mention names, credential, affiliations of the developers, sponsors, and owners**		
**5-ii) Describe the history/development process**		
**5-iii) Revisions and updating**		
**5-iv) Quality assurance methods**		
**5-v) Ensure replicability by publishing the source code, and/or providing screenshots/screen-capture video, and/or providing flowcharts of the algorithms used**		
**5-vi) Digital preservation**		
**5-vii) Access**		
Crash cart APP was free for usual.		
**5-viii) Mode of delivery, features/functionalities/components of the intervention and comparator, and the theoretical framework**		
page 3-4 in methods section.		
**5-ix) Describe use parameters**		
**5-x) Clarify the level of human involvement**		
**5-xi) Report any prompts/reminders used**		
use massage for reminders		
**5-xii) Describe any co-interventions (incl. training/support)**		

page 3 in Study Design Participants and Setting section.		
**6a) CONSORT: Completely defined pre-specified primary and secondary outcome measures, including how and when they were assessed**		
nurse's knowledge and skill		
**6a-i) Online questionnaires: describe if they were validated for online use and apply CHERRIES items to describe how the questionnaires were designed/deployed**		
use questionnaire(SUS, IMMS, CLT, and learning effection questionnaire)		
**6a-ii) Describe whether and how “use” (including intensity of use/dosage) was defined/measured/monitored**		
**6a-iii) Describe whether, how, and when qualitative feedback from participants was obtained**		
**6b) CONSORT: Any changes to trial outcomes after the trial commenced, with reasons**		
page 3 in manuscripts(The research setting was a medical center in southern Taiwan. All nurses were required to have a university degree as part of their work at the institution.)		
**7a) CONSORT: How sample size was determined**		
**7a-i) Describe whether and how expected attrition was taken into account when calculating the sample size**		
in page 4 Participants section.		
**7b) CONSORT: When applicable, explanation of any interim analyses and stopping guidelines**		
nurse's knowledge and skill		
**8a) CONSORT: Method used to generate the random allocation sequence**		
page 3 in methods section		
**8b) CONSORT: Type of randomisation; details of any restriction (such as blocking and block size)**		
in page 4 Participants section.		
**9) CONSORT: Mechanism used to implement the random allocation sequence (such as sequentially numbered containers), describing any steps taken to conceal the sequence until interventions were assigned**		
in page 4 Participants section.		
**10) CONSORT: Who generated the random allocation sequence, who enrolled participants, and who assigned participants to interventions**		
in page 3 setting section.		
**11a) CONSORT: Blinding - If done, who was blinded after assignment to interventions (for example, participants, care providers, those assessing outcomes) and how**		
**11a-i) Specify who was blinded, and who wasn’t**		
data collection in page 5.		
**11a-ii) Discuss e.g., whether participants knew which intervention was the “intervention of interest” and which one was the “comparator”**		
**11b) CONSORT: If relevant, description of the similarity of interventions**		
not applicable(no placebo in this study)		
**12a) CONSORT: Statistical methods used to compare groups for primary and secondary outcomes**		
page 6 in manuscript (results section)		
**12a-i) Imputation techniques to deal with attrition / missing values**		
No participants dropped out.		
**12b) CONSORT: Methods for additional analyses, such as subgroup analyses and adjusted analyses**		
page 7-8(nurse experience less than 2 years)		
RESULTS		
**13a) CONSORT: For each group, the numbers of participants who were randomly assigned, received intended treatment, and were analysed for the primary outcome**		
in page 6 result section.		
**13b) CONSORT: For each group, losses and exclusions after randomisation, together with reasons**		
Data were collected between August and December 2021. No participants dropped out. In total, 102 nurses completed the course, with 43 nurses in the AR group and 59 nurses in the control group.		
**13b-i) Attrition diagram**		
No participants dropped out		
**14a) CONSORT: Dates defining the periods of recruitment and follow-up**		
Data were collected between August and December 2021.		
**14a-i) Indicate if critical “secular events” fell into the study period**		
**14b) CONSORT: Why the trial ended or was stopped (early)**		
participants enough (total 102 nurses in this study)		
**15) CONSORT: A table showing baseline demographic and clinical characteristics for each group**		
page 4 in manscript(A priori power analysis was conducted using G*Power (Heinrich-Heine-Universität Düsseldorf). The sample size (82) was obtained using a power level of 0.8, an α level set at .05, and a small to medium effect size of 0.3 [16,17]. The estimated dropout rate was 20%, and the estimated number of persons to be recruited was approximately 99.)		
**15-i) Report demographics associated with digital divide issues**		
page 6 (Demographic Characteristics section)		
**16a) CONSORT: For each group, number of participants (denominator) included in each analysis and whether the analysis was by original assigned groups**		
**16-i) Report multiple “denominators” and provide definitions**		
page 6 (Demographic Characteristics section)		
**16-ii) Primary analysis should be intent-to-treat**		
**17a) CONSORT: For each primary and secondary outcome, results for each group, and the estimated effect size and its precision (such as 95% confidence interval)**		
data analysis(p-value <0.005 is significance)		
**17a-i) Presentation of process outcomes such as metrics of use and intensity of use**		
**17b) CONSORT: For binary outcomes, presentation of both absolute and relative effect sizes is recommended**		
data analysis(p-value <0.005 is significance)		
**18) CONSORT: Results of any other analyses performed, including subgroup analyses and adjusted analyses, distinguishing pre-specified from exploratory**		
data analysis(p-value <0.005 is significance) in result section		
**18-i) Subgroup analysis of comparing only users**		
page 7-9 result section(Learning outcome, and Learning outcome of crash cart)		
**19) CONSORT: All important harms or unintended effects in each group**		
no harms or unintended effects in each group		
**19-i) Include privacy breaches, technical problems**		
**19-ii) Include qualitative feedback from participants or observations from staff/researchers**		
DISCUSSION		
**20) CONSORT: Trial limitations, addressing sources of potential bias, imprecision, multiplicity of analyses**		

**20-i) Typical limitations in ehealth trials**		
we can not blind for this study.		
**21) CONSORT: Generalisability (external validity, applicability) of the trial findings**		
**21-i) Generalizability to other populations**		
**21-ii) Discuss if there were elements in the RCT that would be different in a routine application setting**		
**22) CONSORT: Interpretation consistent with results, balancing benefits and harms, and considering other relevant evidence**		
**22-i) Restate study questions and summarize the answers suggested by the data, starting with primary outcomes and process outcomes (use)**		
in page 7-9 result section		
**22-ii) Highlight unanswered new questions, suggest future research**		
Other information		
**23) CONSORT: Registration number and name of trial registry**		
Registration: NCT06057285, 09/20/2023, Retrospectively registered		
**24) CONSORT: Where the full trial protocol can be accessed, if available**		
NCT06057285, 09/20/2023, Retrospectively registered		
**25) CONSORT: Sources of funding and other support (such as supply of drugs), role of funders**		
We thank the National Cheng Kung University Hospital (grant no.NCKUH-11008019) and Taiwan's Ministry of Science and Technology (grant no. MOST-109-2511-H-165-002) for their support.		
**X26-i) Comment on ethics committee approval**		
in page 5(this study was approved by the Institutional Review Board of the National Cheng Kung University Hospital (approval A-ER-109-383).)		
**x26-ii) Outline informed consent procedures**		
this study was approved by the Institutional Review Board of the National Cheng Kung University Hospital (approval A-ER-109-383).		
**X26-iii) Safety and security procedures**		
not applicable because all participants have sign agreement before attendance		
**X27-i) State the relation of the study team towards the system being evaluated**		
